# Diversity of tryptophan halogenases in sponges of the genus *Aplysina*

**DOI:** 10.1093/femsec/fiz108

**Published:** 2019-07-05

**Authors:** Johanna Gutleben, Jasper J Koehorst, Kyle McPherson, Shirley Pomponi, René H Wijffels, Hauke Smidt, Detmer Sipkema

**Affiliations:** 1 Laboratory of Microbiology, Wageningen University & Research, Stippeneng 4, 6708 WE, Wageningen, The Netherlands; 2 Laboratory of Systems and Synthetic Biology, Wageningen University & Research, Stippeneng 4, 6708 WE, Wageningen, The Netherlands; 3 Bioprocess Engineering, AlgaePARC, Wageningen University & Research, 6700 AA, Wageningen, The Netherlands; 4 Florida Atlantic University – Harbor Branch, 5600 U.S. 1, Fort Pierce, FL 34946, the United States; 5 Faculty of Biosciences and Aquaculture, Nord University, 8026 Bodø, Norway

**Keywords:** Halogenase, host-associated microbiome, phylogenetic diversity, marine sponges, bioactive compounds, environmental enzymes

## Abstract

Marine sponges are a prolific source of novel enzymes with promising biotechnological potential. Especially halogenases, which are key enzymes in the biosynthesis of brominated and chlorinated secondary metabolites, possess interesting properties towards the production of pharmaceuticals that are often halogenated. In this study we used a polymerase chain reaction (PCR)-based screening to simultaneously examine and compare the richness and diversity of putative tryptophan halogenase protein sequences and bacterial community structures of six *Aplysina* species from the Mediterranean and Caribbean seas. At the phylum level, bacterial community composition was similar amongst all investigated species and predominated by Actinobacteria, Chloroflexi, Cyanobacteria, Gemmatimonadetes, and Proteobacteria. We detected four phylogenetically diverse clades of putative tryptophan halogenase protein sequences, which were only distantly related to previously reported halogenases. The Mediterranean species *Aplysina aerophoba* harbored unique halogenase sequences, of which the most predominant was related to a sponge-associated *Psychrobacter-*derived sequence. In contrast, the Caribbean species shared numerous novel halogenase sequence variants and exhibited a highly similar bacterial community composition at the operational taxonomic unit (OTU) level. Correlations of relative abundances of halogenases with those of bacterial taxa suggest that prominent sponge symbiotic bacteria, including Chloroflexi and Actinobacteria, are putative producers of the detected enzymes and may thus contribute to the chemical defense of their host.

## Teaser

A diverse range of novel halogenase genes was detected in six sponge species, indicating that microorganisms play a fundamental role in the production of sponge-derived bioactive compounds.

## INTRODUCTION

### Bioactive compounds and the role of halogenase enzymes

The need to discover novel drug candidates is high on the policy agenda due to the ongoing emergence of multi-drug resistant microbial strains (O'Neill 2016). The call for a better supply of new drugs against a large range of infectious diseases points towards novel natural products as a yet inexhaustible source of bioactivity. Especially the marine environment proved to be a particularly rich resource for novel bioactive compounds, and many of them are halogenated (Gribble 2015). Carbon–halogen bonds lead to an increase in thermal and oxidative stability as well as increased permeability through biological membranes compared to their non-halogenated counterparts (Smith *et al*. 2017). Halogenated molecules exhibit a wide range of biological activities and may have antifungal, antibacterial, antiviral, anticancer, and/or anti-inflammatory properties (Butler & Sandy 2009; Gribble 2015). Thus, many pharmaceutical and agrochemical compounds as well as polymers are halogenated (Bolton *et al*. 2011; Lu *et al*. 2012; Jeschke 2013), such as the commercially important antibiotics chloramphenicol, vancomycin and teicoplanin (Van Pée and Zehner 2003). In nature, halogens, including chloride, bromide, fluoride or iodide, get attached to organic molecular scaffolds by halogenase enzymes, which have been detected in fungi, bacteria and algae from terrestrial and marine environments (Atashgahi *et al*. 2018; Latham *et al*. 2018). While marine enzymes preferentially halogenate with bromide (Neubauer *et al*. 2018), chlorinated compounds are regularly detected in terrestrial sources (Latham *et al*. 2018). To date, more than 5000 naturally produced halogenated compounds (Gribble 2015) and six independently evolved halogenase enzyme families have been identified (Xu and Wang 2016). These enzyme families, namely cofactor-free haloperoxidase, vanadium-dependent haloperoxidase, heme iron-dependent haloperoxidase, non-heme iron-dependent halogenase, flavin-dependent halogenase, and *S*-adenosyl-l-methionine-dependent halogenase, differ in their cofactor dependence, sequence homology and mechanistic features (Xu and Wang 2016). The most extensively characterized halogenases are flavin (FADH_2_)-dependent halogenases (FDHs) (Van Pée and Patallo 2006; Zhao *et al*. 2015). In contrast to haloperoxidases, FDHs often halogenate regioselectively, and are often part of secondary metabolite gene clusters encoding for non-ribosomal peptide synthetases (NRPS) and polyketide synthetases (PKS)-producing halogenated molecules (Walsh *et al*. 2001; Van Pée and Unversucht 2003; Dorrestein *et al*. 2005; Van Pée and Patallo 2006). Regioselective halogenation under mild reaction conditions without harmful waste generation renders FDHs promising tools for biocatalysis of halogenated compounds (Smith, Grüschow and Goss 2013; Grüschow *et al*. 2015; Shepherd *et al*. 2015; Menon *et al*. 2016; Weichold, Milbredt and Van Pée 2016; Latham *et al*. 2018). The amino acid sequences of FDHs contain a highly conserved flavin binding site (GxGxxG) near the N-terminus, which binds the cofactor in order to create the reactive halogen, as well as a motif with two tryptophan residues (WxWxIP), which presumably prevents the binding of a substrate close to the flavin (Van Pée and Zehner 2003; Van Pée and Patallo 2006). These motifs can be detected in almost every FDH described today, and serve as identification marker for novel FDH genes (Bayer *et al*. 2013). FDHs can be distinguished into three classes depending on their preferred substrate moieties: phenols, pyrroles or tryptophan (Murphy 2006; Van Pée and Patallo 2006). In most cases, the exact substrates for halogenases encoded within biosynthetic gene clusters remain to be identified, however, many of the bacterial FDHs characterized to date halogenate a range of tryptophan derivatives and other aromatic substrates (Payne, Andorfer and Lewis 2013; Frese *et al*. 2014; Shepherd *et al*. 2015). These flavin-dependent tryptophan halogenases (THs) exhibit a broad substrate tolerance for other electron-rich organic scaffolds, although these are halogenated with lower reaction efficiencies in *in vitro* activity tests (Weichold, Milbredt and Van Pée 2016; Agarwal *et al*. 2017). Since halotryptophans occur fairly frequently in natural products (Smith *et al*. 2017), we focused our efforts on THs in this study. Most flavin-dependent THs described to date require the activity of an additional enzyme, a flavin reductase, to provide the reduced flavin cofactor, and are thus two-component systems (Agarwal *et al*. 2017). Single-component halogenating enzymes possess both domains for flavin-reduction and halogenation. To date, only two single-component halogenating enzymes have been reported from marine bacteria, but they remain poorly characterized (Agarwal and Moore 2014; Agarwal *et al*. 2014).

### Sponges as source of novel halogenase sequence variants

Sponges are the most prolific marine invertebrates for the discovery of novel bioactive compounds (Blunt *et al*. 2009; Hu *et al*. 2011; Mehbub *et al*. 2014; Rocha-Martin *et al*. 2014; Sipkema 2017; Carroll *et al*. 2019) since many of them depend on a chemical arsenal to defend themselves against diseases, competitors and predators (Thoms, Ebel and Proksch 2006; Taylor *et al*. 2007; Pawlik 2011). These ancient, filter feeding animals harbour dense and diverse microbial communities including members of the bacterial phyla Actinobacteria, Acidobacteria, Bacteriodetes, Chloroflexi, Cyanobacteria, Planctomycetes, Proteobacteria, Nitrospira, Poribacteria, Tectomicrobia, Verrucomicrobia, as well as archaea and numerous microeukaryotes (Taylor *et al*. 2007; Webster and Taylor 2012; Thomas *et al*. 2016; Chaib De Mares *et al*. 2017). Some of these largely uncultured bacteria can produce highly potent bioactive natural products, many of which are halogenated (Gribble 2010; Smith *et al*. 2017). Amongst marine sponges, the Demosponge genus *Aplysina* represents a morphologically diverse group of species challenging to identify due to their lack of a mineral skeleton (Zea, Henkel and Pawlik 2014), as well as a high degree of sequence conservation in molecular marker genes (Erpenbeck *et al*. 2007; Cruz-Barraza *et al*. 2012). Chemotaxonomy was suggested as additional phylogenetic marker (Erpenbeck and van Soest 2007), since *Aplysina* species are strongly chemically defended and especially renowned for the production of more than 100 halogenated natural products (Lira *et al*. 2011; Loh and Pawlik 2014). Such metabolites can make up to 12% of the sponge dry weight (Turon, Becerro and Uriz 2000; Thoms, Ebel and Proksch 2006).

A survey of natural products from Caribbean *Aplysina* species suggested that the brominated alkaloids were sponge-derived rather than microbiome-derived, since metabolite profiles were highly correlated to sponge morphotype rather than location and depth (Puyana *et al*. 2015). It should be noted, however, that this study did not assess microbial composition, and hence, an essential or auxiliary role of the microbiome in metabolite production cannot be excluded. Halogenated compounds have been reported to be located in spherulous cells of *Aplysina aerophoba*, suggesting production by the sponge, or a complex symbiotic pattern with microorganisms involved at different levels of the biotransformation pathway (Turon, Becerro and Uriz 2000). Significant correlations of the relative abundances of a member of the Chloroflexi, a deltaproteobacterium and an unidentified bacterial OTU with the concentrations of three alkaloids (aerophobin-1, aplysinamisin-1 and isofistularin-3) in *A. aerophoba* indicated that bacteria were correlated to the production of brominated alkaloids (Sacristán-Soriano *et al*. 2011, Sacristán-Soriano, Banaigs and Becerro 2016). This finding corroborates observations that the majority of halogenating enzymes has been described from algae, fungi and bacteria (Xu and Wang 2016). Halogenated natural products or the corresponding biosynthetic gene clusters could previously be associated to specific bacterial symbionts in other marine sponges (Unson, Holland and Faulkner 1994; Flatt *et al*. 2005; Ridley *et al*. 2005; Hochmuth and Piel 2009; Della Sala *et al*. 2013; Öztürk *et al*. 2013; Smith *et al*. 2017), but to the best of our knowledge this remains unresolved for the metabolites of *Aplysina* species, from both the Mediterranean and Caribbean Sea (Puyana *et al*. 2015; Sacristán-Soriano, Banaigs and Becerro 2016). Currently, only dehalogenation mechanisms could be directly linked to the microbial community of *A. aerophoba* (Ahn *et al*. 2003). However, the conserved nature of key enzymes encoded in biosynthetic gene clusters allows the design of degenerate PCR primers, thus facilitating the screening and discovery of novel sequence variants of these enzymes from environmental DNA (Hornung *et al*. 2007; Kennedy, Marchesi and Dobson 2008; Borchert *et al*. 2016). Accordingly, PCR-based surveys have led to the identification of numerous putative flavin-dependent halogenase encoding genes from different environmental samples (Erol, Arends and Muyzer 2017), multiple marine sponges (Bayer *et al*. 2013; Öztürk *et al*. 2013) and cultivated Actinomycetes strains (Hornung *et al*. 2007; Gao and Huang 2009; Liao *et al*. 2016).

### Aim

With this study we aimed to investigate the phylogenetic diversity and distribution of flavin-dependent tryptophan halogenase protein sequence variants within six species of the marine sponge genus *Aplysina*. By using 16S rRNA and TH gene amplicon sequencing, we aimed to determine the resident sponge bacterial community of different *Aplysina* species and explore potential links between the microbial populations and the associated halogenase sequences. This will increase our understanding of the putative producers of halogenated secondary metabolites in sponges.

## MATERIALS and METHODS

### Sponge collection

Three individuals, each of the Caribbean sponge species *Aplysina archeri* (AAr), *Aplysina cauliformis* (ACa), *Aplysina fistularis* (AFi), *Aplysina fulva* (AFu) and *Aplysina lacunosa* (ALa), were collected around Bonaire by SCUBA diving at depths between 1 and 10 m on 8 and 9 October 2012 (Table 1). A sampling permit was given to Detmer Sipkema by the government of Bonaire. Sampled sponge individuals grew at least 1 m apart from each other. The sponge species were identified in the field by Dr. Shirley Pomponi. After sampling, the sponge fragments were rinsed three times with sterile artificial seawater (ASW, 33 g/L Reef Crystals, Blacksburg, VA, USA), cut into 1 cm^3^ pieces and stored in RNAlater (Sigma Aldrich) at −20°C. Additionally, three individuals of the Mediterranean sponge species *Aplysina aerophoba* (AAe) were collected by SCUBA diving at Cala Montgo, Spain (N 42.114, E 3.168), on 19 October 2010 and 15 January 2012. The collection of *A. aerophoba* samples was conducted in strict accordance with Spanish and European regulations within the rules of the Spanish National Research Council with the approval of the Directorate of Research of the Spanish Government. After sampling, the sponges were transported to the laboratory and rinsed three times with sterile artificial seawater (ASW) before grinding with a sterilized mortar and pestle. To obtain a homogenous cell suspension, two volumes of ASW were added. Cell suspensions were aliquoted and mixed with sterile glycerol in ASW for a final concentration of 17% glycerol. Samples were frozen at −20°C and stored at −80°C until DNA extraction (Sipkema *et al*. 2011).

**Table 1.** Metadata, sample information and results of 16S rRNA gene as well as halogenase gene sequencing of the Aplysina species analyzed in this study. THs: tryptophan halogenases. (*) Samples did not pass quality criteria and were excluded from the analyses. Putative halogenase sequences were clustered at the amino acid sequence level.

Sample ID	Species	Sea	Latitude	Longitude	Depth (m)	Temp.	Preservation	16S rRNA genes			Halogenases	
								Reads	OTUs (97%)	Phyla	Reads	Putative THs (95%)
AAe1	*Aplysina aerophoba*	Mediterranean	42.114	3.168	8	*NA*	Cryopreservation	8707	268	17	2813	10
AAe2*	*Aplysina aerophoba*	Mediterranean	42.114	3.168	8	*NA*	Cryopreservation	*NA*	*NA*	*NA*	*NA*	*NA*
AAe3	*Aplysina aerophoba*	Mediterranean	42.115	3.168	12	*NA*	Cryopreservation	6032	241	19	1716	12
AAr1	*Aplysina archeri*	Caribbean	12.160	-68.283	10	28°C	RNALater	9258	312	17	844	8
AAr2	*Aplysina archeri*	Caribbean	12.160	-68.283	10	28°C	RNALater	9786	359	16	2927	18
AAr3	*Aplysina archeri*	Caribbean	12.160	-68.283	10	28°C	RNALater	5397	361	16	1631	17
ACa1	*Aplysina cauliformis*	Caribbean	12.026	-68.251	16	29°C	RNALater	11 664	429	18	907	15
ACa2	*Aplysina cauliformis*	Caribbean	12.026	-68.251	16	29°C	RNALater	5523	341	18	1218	20
ACa3	*Aplysina cauliformis*	Caribbean	12.026	-68.251	16	29°C	RNALater	3890	292	14	1210	17
AFi1	*Aplysina fistularis*	Caribbean	12.094	-68.232	1	30°C	RNALater	4789	223	16	658	11
AFi2	*Aplysina fistularis*	Caribbean	12.094	-68.232	1	30°C	RNALater	8429	181	17	688	11
AFi3*	*Aplysina fistularis*	Caribbean	12.094	-68.232	1	30°C	RNALater	*NA*	*NA*	*NA*	*NA*	*NA*
AFu1	*Aplysina fulva*	Caribbean	12.160	-68.283	10	28°C	RNALater	9995	304	18	5324	12
AFu2	*Aplysina fulva*	Caribbean	12.160	-68.283	10	28°C	RNALater	9988	295	17	2110	11
AFu3	*Aplysina fulva*	Caribbean	12.160	-68.283	10	28°C	RNALater	10 140	424	17	4308	14
ALa1	*Aplysina lacunosa*	Caribbean	12.160	-68.283	10	28°C	RNALater	4896	314	16	2360	15
ALa2	*Aplysina lacunosa*	Caribbean	12.160	-68.283	10	28°C	RNALater	7198	459	18	6976	22
ALa3	*Aplysina lacunosa*	Caribbean	12.160	-68.283	10	28°C	RNALater	6450	282	15	4497	18

### DNA isolation

DNA was extracted using the FastDNA Spinkit for Soil (MP Biochemicals, Santa Ana, CA, USA) according to the manufacturer's instructions with the following modification for the first step: instead of using 500 mg of soil, 750 µl of cryopreserved *Aplysina aerophoba* cell suspension was centrifuged at 14 000 g for 10 min, and the pellet was used for the extraction. For the Caribbean sponge species, 500 mg of wet-weight sponge tissue was rinsed in sterile ASW, cut into small pieces and used for DNA extraction.

### PCR amplification of tryptophan halogenase genes

A PCR-based method was used to screen the sponge samples for the presence and identity of potential flavin-dependent tryptophan halogenase genes. In a preliminary screening among previously reported primers, the degenerate halogenase gene targeted primers SZ002 and SZ005 (Zehner *et al*. 2005) were found the most suitable to amplify an approximately 500 bp DNA fragment. Barcoded PCR amplicons (1 specific barcode for each sample) were obtained through a two-step PCR reaction. For the first PCR reaction, the halogenase primers (SZ002 and SZ005) included linker sequences (341F and 806R): 341F-**SZ002** 5′-CMTAYGGGRBGCASCAG-**TCGGYGTSGGCGARGCGACCRTCCC-**3′ and 806R-**SZ005** 5′-GGACTACNNGGGTATCTAAT-**GCCGGAGCAGTCGAYGAASAGGTC**-3′. The linker sequences represent the binding regions for the barcoding primers in the second PCR. As we routinely use barcoded 16S rRNA gene targeted primers for prokaryotic community composition analysis, we employed those for barcoding of the less routinely used TH gene-targeted primers. Thus, the more widely used barcoding primers could be applied to barcode halogenase gene amplicons.

The first PCR amplification was performed in a volume of 50 µL using 10 µL 5x GoTaq buffer, 2 µL 10 mM dNTP mixture, 0.5 µL 5 U/µL GoTaq DNA polymerase (Promega, Madison, WI, USA), 3 µL 10 µM solution of both primer 341F-SZ002 and primer 806R-SZ005, 22.5 µL nuclease-free water and 1 µL template DNA (10–20 ng/µL) for each of the samples listed in Table 1. PCR conditions were initial denaturation (94°C for 5 min), followed by 35 cycles of denaturation (94°C for 30 s), annealing (60°C for 40 s), elongation (72°C for 50 s) and a final extension (72°C for 5 min). Amplification products were visualized on a 1.25% (w/v) agarose gel and purified using the Millipore DNA Gel Extraction Kit (Millipore, Billerica, MA, USA). A second barcoding PCR was performed as described earlier, except that a pyrosequencing adapter A (CCATCTCATCCCTGCGTGTCTCCGACTCAG) and 18 different barcodes of 10 nucleotides length connected to the 341F linker sequence were used as forward primer and pyrosequencing adapter B (CCTATCCCCTGTGTGCCTTGGCAGTCTCAG) connected to the 806R linker sequence as reverse primer. Furthermore, the number of amplification cycles was reduced to 15. PCR products were visualized on a 1% (w/v) agarose gel, and the bands of PCR products were excised from the gel and purified as described earlier. The amplified fragments with adapter and barcodes were quantified using a Qubit fluorometer (Invitrogen) and mixed in approximately equal concentrations (4 × 10^5^ copies μL^−1^) to ensure equal representation of each sample in the pool. A 454-sequencing run was performed on a GS FLX Standard PicoTiterPlate (70 × 75) using a GS FLX pyrosequencing system according to the manufacturer's instructions (Roche, Mannheim, Germany) at the Technical University of Copenhagen. Pyrosequencing data of halogenase genes were deposited at the NCBI Sequence Read Archive under sample accession numbers SRR7853828–SRR7853845.

### PCR amplification of 16S rRNA genes

Barcoded amplicons of bacterial 16S rRNA genes for all sponge samples were amplified from the extracted DNA. PCR reactions were performed in a volume of 100 μL containing 20 μL High Fidelity Buffer (ThermoFisher Scientific, Waltham, MA, USA), 2.5 μL 10 μM 338R-I, 2.5 μL 10 μM 338R-II reverse primer (Daims *et al*. 1999), 2 μL 10 mM dNTP mixture, 1 μL 2 U/μL Phusion Hot start II DNA polymerase, 65 μL nuclease free water. 5 µl 27F-DegS forward primer (van den Bogert *et al*. 2011) with Titanium adapter A and a sample-specific barcode (8nt) (Hamady *et al*. 2008) attached to the 5′- end as well as 2 µL template DNA (10–20 ng/µL) were added to each reaction. Amplification conditions were initial denaturation (98°C for 30 s), followed by 30 cycles of denaturation (98°C for 10 s), annealing (56°C for 20 s), elongation (72°C for 20 s) and a final extension (72°C for 10 min). Amplification products were visualized, purified, pooled and sequenced as described earlier. Pyrosequencing data of 16S rRNA genes were deposited at the NCBI Sequence Read Archive under sample accession numbers SRR7853935–SRR7853950.

### Halogenase gene amplicon data analysis

Halogenase gene pyrosequencing data were demultiplexed using QIIME version 1.9.0 (Caporaso *et al*. 2010). Sequences that (i) were shorter than 200 bp or longer than 1000 bp, (ii) contained more than one mismatch in the forward or reverse primer sequences, (iii) contained ambiguous bases or (iv) were represented with less than three reads were removed. Chimeric sequences were detected using the usearch61 algorithm (Edgar 2010) and removed. Two samples (AAe2 and AFi3) did not pass quality criteria of the 16S rRNA gene data analysis and were thus also removed from the halogenase gene analyses. The remaining sequences were translated into the three forward open reading frames using the transeq algorithm (Blankenberg *et al*. 2007) as implemented in Galaxy (Afgan *et al*. 2016), and ORFs containing stop codons were removed using customized Bash and R scripts (https://github.com/mibwurrepo/Gutleben_et.al_Halogenases_Aplysinas). Sequences were clustered at 95% amino acid sequence identity based on the average protein sequence identity in genomes of the same bacterial species (Rodriguez-R and Konstantinidis 2014; Chaib De Mares *et al*. 2018) using the uclust algorithm (Edgar 2010). The most abundant sequence per cluster was retained as representative sequence.

For identification of putative flavin-dependent TH sequences, a reference database was created, by subsetting the UniProt/SwissProt database (Bairoch 2002; Bateman *et al*. 2017) to 5427 ‘halogenase’ protein entries, since similarity searches to smaller databases return more sensitive results (Jagtap *et al*. 2013; Pearson 2014). Of these, 75 were manually annotated and reviewed entries (SwissProt). Representative amino acid sequences were aligned (blastp) against (i) the entire UniProt/SwissProt database (release 2018_02) and (ii) the halogenase database using the Diamond alignment tool (Buchfink *et al*. 2014). Protein families (Pfam) were assigned using the InterProScan pipeline 5.17 (Quevillon *et al*. 2005) based on an evalue cutoff of 10^−6^.

Amino acid sequences that aligned significantly (*e*-value < 0.001; Pearson 2014) against an entry in the halogenase database were retained for phylogenetic analyses. Sequences were aligned using the ClustalW algorithm (Larkin *et al*. 2007), together with the most closely related database entries, four reference sequences (tryptophan 5-halogenase PyrH (*Streptomyces rugosporus*, A4D0H5), flavin-dependent TH RebH (*Lechevalieria aerocolonigenes*, Q8KHZ8), flavin-dependent TH PrnA (*Pseudomonas fluorescens*, P95480), halogenase ClaH (*Streptomyces uncialis*, G3K6J6)), putative TH protein sequences previously found in the sponge *C. crambe* (Öztürk *et al*. 2013) and the outgroup sequences NADH-dependent flavin oxidoreductase BaiH (*Clostridium scindens*, P32370) and NADPH-flavin oxidoreductase Frp (*Vibrio harveyi*, Q56691). The halogenase sequences from *A. aerophoba* obtained by Bayer *et al*. (2013) could not be included in this analysis since they covered a different region of the gene.

The resulting alignment was manually refined and trimmed to the amplified regions excluding the primers using Jalview (Waterhouse *et al*. 2009). A maximum likelihood phylogenetic tree was calculated using RaXML HPC (Stamatakis 2006) under the PROTGAMMAWAG substitution model, and 100 bootstrap replicates were used to evaluate clusters. The best-scoring tree was visualized using iTol (Letunic and Bork 2016).

R (version 3.4.3) (Sasaki, Massaki and Kubo 2005) was used for diversity analyses of amino acid sequences identified as putative tryptophan halogenases. For visualization and interpretation, relative abundance information was used for interpretation and was visualized using the ggplot2 v.2.2.1 package (Wickham 2016). Weighted UniFrac dissimilarities (Lozupone *et al*. 2011) were calculated and ordinated using Principal Coordinates Analysis as implemented in the phyloseq package (McMurdie and Holmes 2013). Phyloseq and the microbiome package (Lahti *et al*. 2017) were used for calculating observed richness and Shannon index diversity. Faith's phylogenetic diversity was calculated using the package picante (Kembel *et al*. 2010). Venn diagrams were calculated and visualized using online tool jvenn (Bourtzis *et al*. 1996). Caribbean core halogenases were defined as being present in at least one sample from all the Caribbean species and identified using jvenn.

### 16S rRNA gene amplicon data analysis

Bacterial 16S rRNA gene pyrosequencing data were analyzed using mothur v.1.39.5 (P. D. Schloss *et al*. 2009) by following the 454 Standard operating procedure (https://www.mothur.org/wiki/454_SOP). In brief, sequences were demultiplexed, denoised, and sequences with (i) more than two mismatches in the primers, (ii) more than one mismatch in the barcode and (iii) more than 8 homopolymer were discarded (*trim.flows* and *trim.seqs* commands). Reads were reduced to unique sequences (*unique.seqs*) and aligned to the SILVA SSU 128 database (Quast *et al*. 2013) (*align.seqs*: flip = t). Aligned reads were kept (*screen.seqs:* optimize = start-end, criteria = 98, minlength = 250), and empty alignment columns were removed (*filter.seqs*: vertical = T, trump = .). Read counts for sequences that were within ≥99% sequence similarity to a more abundant sequence were merged (*pre.cluster:* diffs = 2). Chimeric sequences were detected with Vsearch (*chimera.vsearch*) (Rognes *et al*. 2016) and removed (*remove.seqs*). Taxonomy was assigned using the SILVA SSU 128 database (*classify.seqs:* cutoff = 80) (Wang *et al*. 2007). Sequences that were not classified at Domain level as well as chloroplast sequences were removed (*remove.lineage*). Uncorrected pairwise distances between aligned sequences were calculated (*dist.seqs*: cutoff = 0.15), OTUs were generated on the basis of 97% sequence identity (*cluster:* method = opti, cutoff = 0.03), and files were converted to .shared format (*make.shared:* list = , group = ,). Taxonomy was assigned to OTUs (*classify.otu*: list = , name = , taxonomy = , label = 0.03), and representative sequences for each OTU were picked (*get.oturep*: phylip = , list = , fasta = , label = 0.03, sorted = size). Further OTU table processing was done with Bash and R scripts (https://github.com/mibwurrepo/Gutleben_et.al_Halogenases_Aplysinas). Relative abundance information was used for visualization and interpretation. Calculation of community metrics and UniFrac dissimilarities were performed as described earlier.

Core taxa were defined as being present in at least one sample from all analyzed sponge species, or from all Caribbean sponge species (Caribbean core) and were identified using jvenn. Correlations between relative halogenase and bacterial abundances were expressed as Spearman coefficients for all taxa and all halogenase genes, as well as for the Caribbean core taxa and core halogenase genes. Only coefficients >±0.5 and with *P* < 0.05 were considered significant, and only taxa and halogenases shared by all Caribbean species were included in this analysis to maximize statistical power. Heatmaps were generated using the pheatmap v1.0.8 package (Kolde 2012). Analyses are available as R Markdown (https://github.com/mibwurrepo/Gutleben_et.al_Halogenases_Aplysinas).

## RESULTS

### Identification of putative tryptophan halogenases

To capture the diversity of PCR-amplified halogenase genes in *Aplysina* species, 454-pyrosequencing was performed. A total of 37 374 DNA reads, representing 3653 unique protein sequences, were obtained from TH gene-targeted amplicon sequencing. After clustering the unique sequences at 95% amino acid sequence identity, 1918 protein sequence clusters were retained with a maximum of 109 sequences per cluster. Detailed results per sample are given in Table 1.

Out of the representative (most abundant) sequences of these 1918 clusters, 1654 (86.24%) resulted in a hit against the entire UniProt protein database. However, only 40 sequences aligned significantly (*e*-value <0.001; Pearson 2014) against two flavin-dependent TH entries present in the UniProt database with low sequence identities (<45% amino acid identity) and low bitscores (mean = 56). Thus, the 1918 sequences were blasted against a manually curated ‘halogenase’ protein database containing the halogenase protein sequence subset (5427 entries) from UniProt. In total, 86 sequences (4.5%) resulted in a significant (*e*-value <0.001) hit against 1 of 16 entries from the ‘halogenase’ database with high amino acid sequence similarities (32.9–100%) and high bitscores (mean = 173; Table 2) and were thus identified as putative TH protein sequence fragments. Of these 86 sequences, 25 had 67–80% amino acid sequence identity to a TH from marine gammaproteobacterium HTCC2080, an abundant oligotrophic marine microorganism belonging to the NOR5/OM60 clade (Cho and Giovannoni 2004; Thrash *et al*. 2010). Another 19 sequences matched most closely (82–100%) to a TH fragment from *Psychrobacter* sp. D8, a gammaproteobacterium isolated from the sponge *Crambe crambe* (Öztürk *et al*. 2013). The closest database match of another 15 sequences, although with lower (36–51%) amino acid sequence identity, was a TH from the cyanobacterium *Calothrix* sp. NIES-2100 (Hirose *et al*. 2017).

**Table 2.** Hit table (blastp) of sequences aligned against the manually curated halogenase protein database. *TH: Tryptophan halogenase, THf: Tryptophan halogenase (Fragment). %ID is given as % amino acid sequence identity.

No. of sequences	Closest match UniProt accession	Protein name	Organism	%ID min.	%ID max.	Bitscore avg.	Publication
25	A0Z0U8	TH	marine Gammaproteobacterium HTCC2080	67.3	80.4	223	(Thrash *et al*. 2010)
19	K7W8V2	THf	*Psychrobacter* sp. D8	82.1	100	194	(Öztürk *et al*. 2013)
15	A0A1Z4GVL5	TH	*Calothrix* sp. NIES-2100	36.2	51.2	116	(Hirose *et al*. 2017)
7	A0A2E9M8J4	TH	Dehalococcoidales bacterium	55.9	76.2	169	(Tully, Graham and Heidelberg 2018)
5	A0A1Z4LA92	TH	*Nostoc linckia* NIES-25	38.2	39.9	118	(Tully, Graham and Heidelberg 2018)
3	A0A2D9NM13	TH	Halieaceae bacterium	71.2	83.9	172	(Tully, Graham and Heidelberg 2018)
3	A0A2E6EA89	TH	*Woeseia* sp.	75.5	76.2	245	(Bagnoud *et al*. 2015)
1	A0A0F2QFJ7	TH	Hyphomonadaceae bacterium BRH_c29	67.7	67.7	84	(Bagnoud *et al*. 2015)
1	A0A0F5Q235	TH	*Devosia psychrophila*	36.2	36.2	93	(Lepp *et al*. 2015)
1	A0A0J7XXQ8	TH	*Novosphingobium barchaimii* LL02	32.9	32.9	46	(Pearce, Oakeshott and Pandey 2015)
1	A0A0M4LWL3	TH	*Altererythrobacter epoxidivorans*	42.6	42.6	48	(Li *et al*. 2016)
1	A0A1M6AX62	TH	*Rubritalea squalenifaciens* DSM 18 772	37.9	37.9	100	(Varghese and Submissions 2016)
1	A0A2D9ICU6	TH	*Citromicrobium* sp.	45.5	45.5	41	(Tully, Graham and Heidelberg 2018)
1	A0A2E1UEX3	TH	Gammaproteobacteria bacterium	80.7	80.7	200	(Tully, Graham and Heidelberg 2018)
1	Q9RPF9	TH	*Myxococcus fulvus*	53.9	53.9	182	(Hammer *et al*. 1999)
1	T1WAM0	TH	Uncultured organism	39.5	39.5	48	(Nyyssönen *et al*. 2013)

Additionally, the 86 protein sequences with significant ‘halogenase’ database hits were also investigated for protein domains using InterProScan (Finn *et al*. 2017). This confirmed for 84 sequences that they belong to the ‘Tryptophan halogenase’ protein family (PF04820) and all contained a flavin-TH domain (IPR006905). Only two short protein sequences (S1691 and S1792), which were identified as TH by blastp, did not contain a detectable domain.

### Phylogenetic analyses of putative tryptophan halogenases

**Figure 1.**
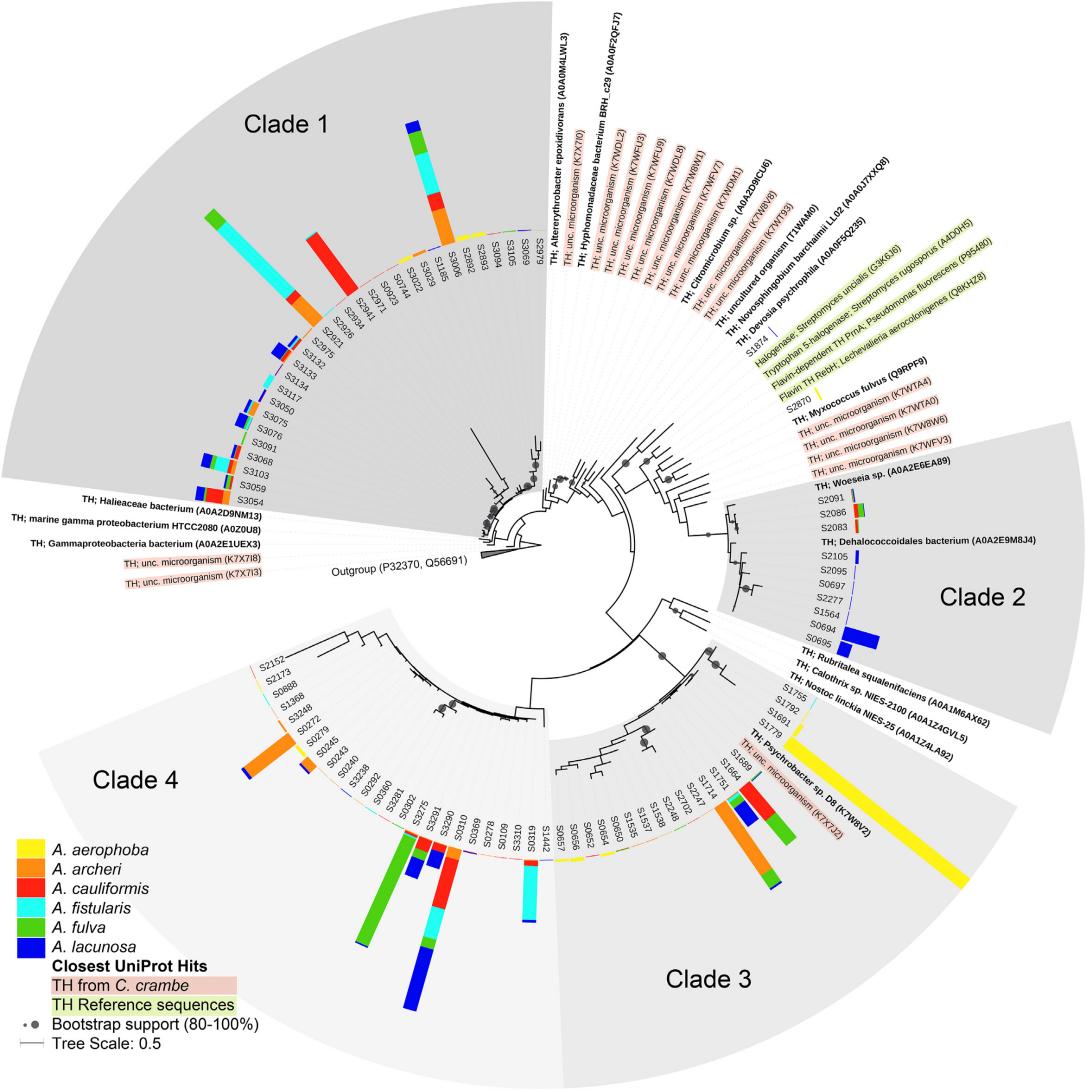
Maximum likelihood phylogeny of putative tryptophan halogenase (TH) protein sequences from six Aplysina species. Sequences obtained in this study are labelled with S. The tree was constructed from 247 amino acid positions. Two NADH-dependent flavin oxidoreductases (P32370 from Clostridium scindens and Q56691 from Vibrio harveyi) were used as outgroup. Four well-studied TH obtained from UniProt (green) and putative TH from the sponge Crambe crambe (pink) are included as reference sequences. Bars indicate relative abundance of the sequences in the sponge species, with the outer rim of the grey shading indicating 88%. Bootstrap values >80% are indicated by grey circles at the branch points. Sequences in bold refer to the closest relatives from the ‘halogenase’ database. UniProt sequence accession numbers are given inside brackets. Tree scale corresponds to the mean expected number of amino acid substitutions per site.

Phylogenetic analyses of the 86 identified putative TH protein sequences resulted in four distinct clades, and only two sequences (S1874 and S2870) did not cluster within these four clades (Fig. 1). Except for *A. aerophoba*, all analyzed sponge species contained halogenases from all four phylogenetic clades. The largest clade (Clade 1) contained 30 sequences, followed by Clade 4 (25 sequences), Clade 3 (19 sequences) and Clade 2 (10 sequences).

Only one species-specific halogenase clade could be identified, namely an *A. lacunosa*-specific subclade within Clade 2 (7 out of 10 sequences; Fig. 1). The sequences within this subclade were all most closely related to a TH from a Dehalococcoidales bacterium (A0A2E9M8J4). Within Clade 3 (19 sequences), the halogenase most abundant (88%) in *A. aerophoba* (S1779) was closely related to a putative halogenase sequence (K7XJ2) previously detected in Mediterranean *C. crambe* as well as to a halogenase from *Psychrobacter*sp. D8 isolated from the same sponge (Öztürk *et al*. 2013). Especially Clade 4 (25 sequences) was only distantly related to previously reported halogenases from Verrucomicrobia and Cyanobacteria and contained mainly sequences from Caribbean *Aplysina* spp., indicating a large number of novel halogenases within these sponges and their microbiota. The well-characterized flavin-dependent TH gene products RebH (*L. aerocolonigenes*, Q8KHZ8; Yeh *et al*. 2007) and PrnA (*P. fluorescens*, P95480; Dong *et al*. 2005) clustered outside the sponge-derived clades and were closely related to only one not abundant (<1%) sequence from *A. aerophoba* (S2870). While the Caribbean species shared many halogenases, it is noteworthy that sequences from the Mediterranean *A. aerophoba* were closely related to Caribbean halogenase sequences, albeit in no case identical. Furthermore it could be observed that some halogenases were highly abundant in only one sponge species such as S2941 in *A. cauliformis* or S3275 in *A. fulva*, while others such as S0310 or S3006 were detected in similar abundances in all Caribbean species.

### Bacterial and halogenase diversity

A total of 122 142 high-quality bacterial 16S rRNA gene sequences were obtained from all sponge samples. These sequences clustered into 1993 OTUs at 97% sequence similarity. Bacterial composition at phylum level was similar for all *Aplysina* species, with Acidobacteria, Actinobacteria, Chloroflexi, Cyanobacteria, Gemmatimonadetes, SBR1093 and Proteobacteria representing the most predominant out of 20 detected bacterial phyla (Supplementary Fig. S1). The 50 overall most abundant OTUs cumulatively accounted for a relative abundance between 59% (ALa2) and 90% (AFi2) in individual samples (Supplementary Fig. S2). Each sponge species exhibited a large number of unique OTUs, while 39 OTUs were shared between all species. These core OTUs comprised between 6% and 45% relative abundance in the sponge samples and contained seawater-derived clades, such as Chloroflexi SAR202 (Morris *et al*. 2004), as well as putative sponge-symbiotic bacteria such as Rhodospirillales (Karimi *et al*. 2018), Acidobacteria and *Nitrospira* (Schmitt *et al*. 2012).

**Figure 2.**
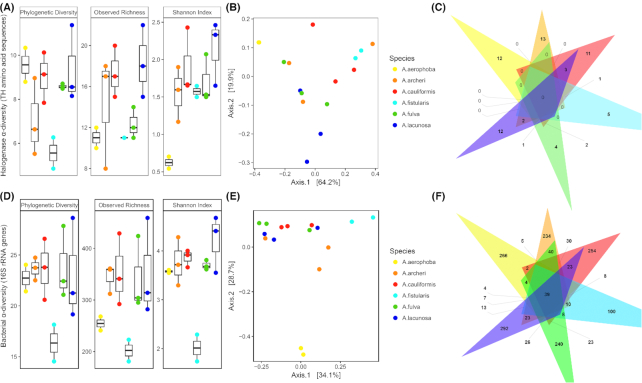
Halogenase and bacterial 16S rRNA gene alpha diversity indices (A,D). PCoA analysis of weighted UniFrac distances on relative abundance information of tryptophan halogenase (TH) amino acid sequences (B) and 16S rRNA genes (E). A. aerophoba replicates are almost identical and thus overlap in plot B. Venn diagrams of shared and unique TH sequences (C) and 16S rRNA genes (F) per sponge species.

All Caribbean *Aplysina* species shared 85 OTUs, amongst which were a predominant sponge-associated member of the Cyanobacteria (Otu0001, 1–67% relative abundance in individual samples), an unclassified bacterial OTU (Otu0010, 0.6–8.2% relative abundance), a member of the actinobacterial OM1 clade (Otu0007, 0.6–8.2% relative abundance) and an SBR1093 OTU (Otu0019, 0.5–4.3% relative abundance). In total, 37 of the 50 overall most abundant OTUs were shared by all of the Caribbean species. Due to the large overlap in bacterial community structure of the Caribbean species, only *A. fistularis* clearly separated from the other Caribbean *Aplysina* spp. due to the high relative abundance of the cyanobacterial Otu0001 (>63% relative abundance). In addition, the Mediterranean *A. aerophoba* could be clearly separated from the other species in ordination plots (Fig. 2E).

In order to investigate connections between the halogenase diversity and the sponge-associated bacterial diversity, alpha and beta diversity indices were calculated for the 86 putative halogenase sequences and the trends were compared to the diversity indices calculated for the bacterial 16S rRNA gene sequences retrieved from the *Aplysina* samples (Fig. 2 and Supplementary Fig. S3). Phylogenetic Diversity, Observed Richness and Shannon Diversity indices for halogenases and bacterial 16S rRNA gene sequences followed a similar trend for all species (Fig. 2A and D), however, correlations of diversity indices were only significant for Observed Richness (Supplementary Fig. S3). Overall, a high phylogenetic diversity of both bacterial OTUs and halogenase sequences was detected for *A. aerophoba*, *A. cauliformis* and *A. lacunosa*, while *A. fistularis* exhibited the least phylogenetically diverse gene repertoire (Fig. 2A). The observed richness of *A. aerophoba* for both halogenases and bacterial 16S rRNA gene sequences was lower than that for the Caribbean species, however the phylogenetic diversity of both measured variables was high, indicating a large phylogenetic breadth. These differences were not statistically significant (anova *P*_adj_> 0.05). Beta diversity analyses showed no discernible species separation as well as a large spread in ordination space for the Caribbean species, while the *A. aerophoba* samples clustered apart from the other species and extremely close to each other, indicating an almost identical halogenase profile as well as bacterial 16S rRNA gene profile (Fig. 2B and E). Overall, the first two axes explained 84% of variation in the halogenase dataset, and 63% in the 16S rRNA gene dataset, where all Caribbean species spread along the first axis, separating only *A. aerophoba* along the second axis (Fig. 2E). This analysis revealed an overlap in the halogenase composition of the Caribbean sponges, who shared four halogenase sequences (S3006, S0310, S3103, and S3275). Additionally, each sponge species harbored between 4 (*A. fulva)* and 13 (*A. archeri)* species-specific halogenase sequences (Fig. 2C).

### Co-correlation of bacterial 16S rRNA gene and halogenase relative abundances

**Figure 3.**
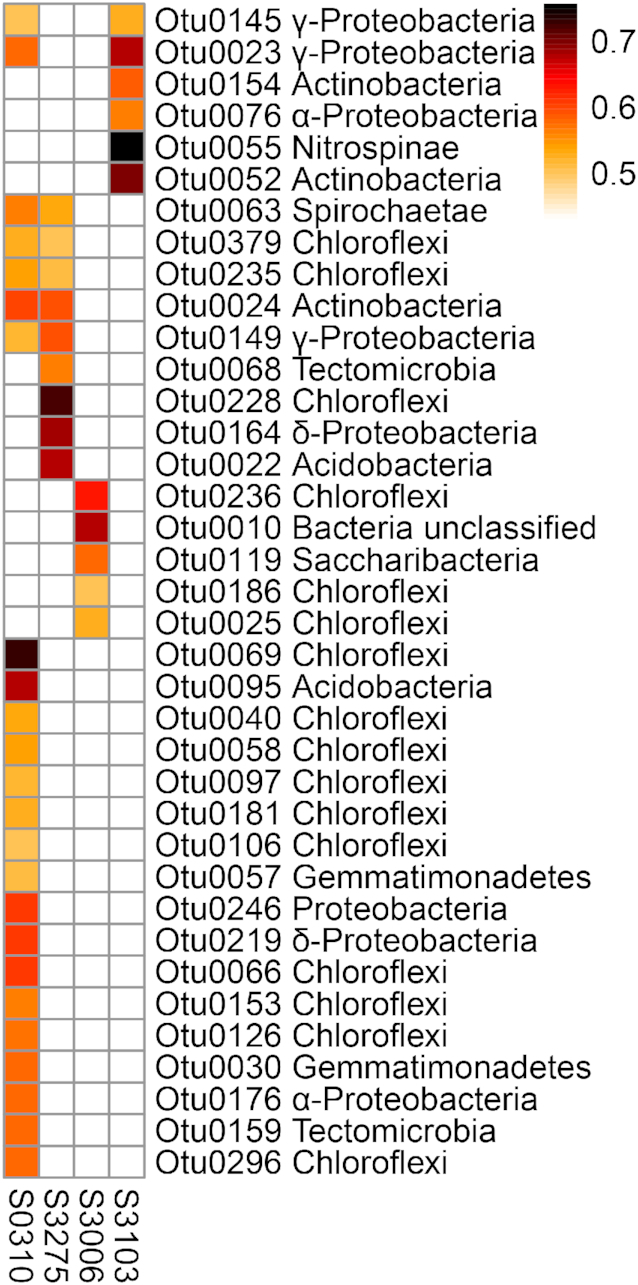
Heatmap displaying significant (Spearman *r* > 0.5, *P*< 0.05) co-occurance correlations of tryptophan halogenases (columns) and bacterial 16S rRNA gene OTUs (rows) shared between all Caribbean Aplysina species. OTU IDs and phyla are given to identify bacterial taxa. Columns and rows are clustered by Euclidian distance. Colors indicate correlation coefficients ranging from 0.5 (orange) to 1 (black).

Since diversity calculations indicated an interrelation between halogenase and bacterial 16S rRNA gene diversity, Spearman correlations on the relative abundances of halogenase amino acid sequences with bacterial 16S rRNA genes were calculated for the 50 most predominant bacterial OTUs. All 50 most predominant OTUs exhibited significant (*P*< 0.05, Spearman *r* > ±0.5) correlations with two or more of the 86 putative halogenases (Supplementary Fig. S4). It should be noted, however, that the large number of unique and distinct halogenases as well as bacterial OTUs derived from the Mediterranean species *A. aerophoba* caused a prominent block of strong correlations (Supplementary Fig. S4), and these results should thus be interpreted carefully. Therefore, we further evaluated only the relative abundance co-correlations of bacterial 16S rRNA genes and halogenases that were shared between all Caribbean species (Fig. 3).

Of the 85 shared bacterial OTUs, 37 were significantly (*P* < 0.05, Spearman r>0.5) positively correlated with the four shared putative halogenases. The shared bacterial OTUs constituted between 28% and 80% (mean = 46%) of the total relative abundance per sample, while the shared halogenases represent between 10% and 71% (mean = 30%) relative abundance. The majority of the correlated OTUs were affiliated to the phyla Chloroflexi (16 OTUs), Proteobacteria (8 OTUs, Alpha-, Delta- and Gamma-proteobacteria), Actinobacteria (3 OTUs) and Tectomicrobia (2 OTUs) (Fig. 3), all of which are renown for the production of halogenating enzymes (Bayer et al. 2013, 2018; Öztürk *et al*. 2013; Liao *et al*. 2016; Smith *et al*. 2017). For three halogenases, one specific bacterial OTU was found especially highly correlated (*r*> 0.72), suggesting this distinct bacterial taxon as potential halogenase producer: The highly predominant halogenases S0310 and S3275, both phylogenetically associated with clade 4, were suggested to be produced by the Chloroflexi Otu0069, member of the sponge-associated lineage TK10 (Schmitt *et al*. 2011; Burgsdorf *et al*. 2014), and an unclassified Chloroflexi Otu0228 with 98% 16S rRNA gene sequence identity to a sponge-associated member of the Chloroflexi (GenBank FJ481334, (Montalvo and Hill 2011)), respectively. The less abundant halogenase S3103, phylogenetically placed in clade 1, was most highly correlated to an unclassified Nitrospinae Otu0055, displaying 97% 16S rRNA gene sequence identity to an uncultured sponge-associated bacterium (GenBank FJ900348; Kamke *et al*. 2010). Despite this analysis being of only predictive nature, the co-occurrence of certain halogenases with specific bacterial taxa can narrow down the range for searching potential microbial producers of these proteins.

## DISCUSSION

The marine environment is a prolific source of novel enzymes with potential industrial applications. To screen marine samples for such enzymes, metagenomics approaches are promising, whereby all extracted DNA is investigated with DNA sequence-based methods (Hugenholtz and Tyson 2008; Kennedy, Marchesi and Dobson 2008; Vakhlu *et al*. 2008; Wilson and Piel 2013; Barone *et al*. 2014; Loureiro *et al*. 2018). For genes where suitable PCR primers can be designed, a PCR-based screening approach is especially useful to rapidly explore diversity of the gene of interest in a larger number of environmental samples. Such approaches have previously been applied for the discovery of novel natural product biosynthesis genes (Zhao, Yang and Zeng 2008; Milshteyn, Schneider and Brady 2014; Amos *et al*. 2015; Müller *et al*. 2015; Borchert *et al*. 2016), lipases (Wang *et al*. 2010) or alcohol dehydrogenases (Itoh, Kariya and Kurokawa, 2014), to just name a few examples from a broad range of functional genes discovered in environmental samples (Kotik, 2009). In this study, the PCR-based screening approach resulted in the discovery of a large phylogenetic breadth of previously undescribed, putative halogenase protein fragments, which may be involved in the biosynthesis of numerous brominated natural products found in Mediterranean and Caribbean *Aplysina* species.

### Sponges harbor numerous novel putative tryptophan halogenases

Out of all detected protein sequences clustered at 95% amino acid sequence identity, 86.24% had a match in the entire UniProt database, hinting at still a large number of unknown protein sequences in marine sponges. Additionally, only 86 out of 1918 sequences could successfully be identified as putative halogenases. This could indicate a low specificity of the primers applied, which is a regularly observed problem for primers targeting functional genes in microbial communities (Mohamed *et al*. 2010; Pereyra *et al*. 2010; Gaby and Buckley, 2012; Bonilla-Rosso *et al*. 2016) due to relatively low degrees of sequence conservation in comparison to the 16S rRNA gene.

On the other hand, the number (86) of putative THs is similar for sponges and other environmental samples such as freshwater, marine and soil environments or cultivated Actinomycetes strains (18 (Öztürk *et al*. 2013), 36 (Bayer *et al*. 2013), 38 (Erol, Arends and Muyzer, 2017), 103 (Hornung *et al*. 2007), 254 (Neubauer *et al*. 2018)). The low sequence identities (mean = 44%), and the match to only few (16) ‘halogenase’ entries from the UniProt database, further indicate a large, yet untapped resource of halogenases in marine sponges. Only one sequence (S2870 in *A. aerophoba*) with low abundance (<1%) was related with <44% amino acid sequence similarity to two well-charaterized flavin-dependent THs, PrnA and RebH (Fig. 1). PrnA is encoded in the biosynthetic gene cluster for the production of the antifungal compound pyrrolnitrin in *Pseudomonas fluorescens* (Harris *et al*. 1985), wheras RebH is part of the biosynthetic gene cluster for the anticancer compound rebeccamycin in *Lechevalieria aerocolonigenes* (Onaka *et al*. 2003).

Due to low sequence identies to well-studied halogenases, the putative THs could not be confidentially assigned to any of the known structural classes of halogenated compounds. These enzymes probably represent novel types, which halogenate different structural moieties compared to previously reported halogenases. Such a TH with an unusual substrate preference has been discovered in the metagenome of the sponge symbiont *Candidatus* Entotheonella serta (Smith *et al*. 2017). This halogenase displays only between 5% and 27% amino acid sequence identity to the halogenases from this study (data not shown) and represents another recent example of the large, yet untapped genetic resources for novel halogenases in marine sponges. Our phylogenetic analyses resulted in four distinct clades, potentially separating functionally divergent groups of halogenases. These findings expand on previously reported results for *A. aerophoba* (Bayer *et al*. 2013) and *C. crambe* (Öztürk *et al*. 2013), which showed three distinct sponge-specific clades of halogenases. Each analyzed sponge species harbored sequences from all four clades, hinting at a potentially highly diverse spectrum of halogenated molecules that can be synthesized within each sponge. Rua *et al*. (2018) hypothesized that larger microbiome diversity influences the potential of bioactive compound production in sponges. In our study we found that a high phylogenetic diversity and richness of microbial taxa corresponded to a high diversity of halogenases. The Mediterranean *A. aerophoba* harbored a bacterial community distinct from that of the Caribbean species, which is consistent with previous investigations (Thomas *et al*. 2016; Chaib De Mares *et al*. 2017), and while its halogenases were closely related to Caribbean ones, they were in no case identical.

All except for two of the detected novel putative halogenases as well as their closest relatives exhibited active binding sites for flavin and l-tryptophan. Since these enzymes are known to tolerate a wide range of organic scaffolds (Agarwal *et al*. 2017) and since all FDHs known to date can also function as brominases (Xu and Wang, 2016), they are potentially halogenating completely unknown organic substrates. Thus, these enzymes may contribute to the production of the more than 100 halogenated natural products reported from *Aplysina* species (Turon, Becerro and Uriz, 2000; Thoms, Ebel and Proksch, 2006; Lira *et al*. 2011; Loh and Pawlik, 2014; Puyana *et al*. 2015). However, further studies are necessary to unravel the exact functions of these enzymes as no closely related enzymes have been functionally characterized (Fig. 1).

### Prominent sponge symbionts are potential halogenase producers

In an attempt to predict the potential bacterial producers of the detected halogenase genes, we correlated the relative abundances of the four 16S rRNA gene OTUs and the 85 putative halogenases shared by the Caribbean *Aplysina* species. We hypothesized that the shared halogenases are produced by bacteria that can be found amongst the shared bacterial taxa in the different sponge species. Furthermore, an increased relative abundance of the producers is expected to be reflected in an increased number of detected halogenase genes. Two halogenases (S0310 and S3275) were most highly correlated with sponge-associated Chloroflexi (Otu0069 and Otu0228, respectively; Fig. 3). These clades include predominant sponge symbionts with the genomic repertoire for chemical defense (Slaby *et al*. 2017; Bayer *et al*. 2018). Chloroflexi were previously found to contain halogenases (Bayer *et al*. 2013) and were linked to the production of brominated compounds in *A. aerophoba* (Sacristán-Soriano *et al*. 2011, Sacristán-Soriano, Banaigs and Becerro 2016). We furthermore detected strong correlation of a halogenase sequence to a Nitrospinae OTU, however little is known about this taxon in sponges to date. Nonetheless, Nitrospinae are closely related to the candidate phylum Tectomicrobia, which occurs in *Aplysina* species (Chaib De Mares *et al*. 2018) and of which some members are renowned for their large secondary metabolism gene repertoire (Wilson *et al*. 2014; Smith *et al*. 2017).

One highly predominant cyanobacterial OTU was present in all Caribbean species and was not significantly correlated to halogenases in this study, adding to the hypothesis that the role of Cyanobacteria in sponge-microbe symbioses of high microbial abundance sponges might be mainly related to nutrient production rather than chemical defense (Freeman *et al*. 2013; Burgsdorf *et al*. 2015). In low microbial abundance sponges, however, the production of brominated metabolites has been linked to cyanobacterial symbionts (Unson, Holland and Faulkner, 1994; Flatt *et al*. 2005). Thus, our results support previous findings and for the first time indicate potential microbial producers of brominated compounds found in Caribbean *Aplysina* species. However, further studies, including comparative and functinoal genomics of sponge symbionts, are necessary to reliably link halogenated compound production to their microbial producers.

## CONCLUSION

The plethora of previously undescribed putative flavin-dependent THs from the metagenomic DNA of Mediterranean and Caribbean *Aplysina* species unraveled here indicates a large potential for the discovery of novel halogenating enzymes from these marine invertebrates and their associated microbiomes. The separation into four phylogenetically distinct clades of halogenase protein sequences indicates that multiple classes of organic scaffolds may be halogenated by sponge-associated microbes. High bacterial diversity was in most cases indicative of a high halogenase diversity, and while the Caribbean species shared many halogenases as well as bacterial OTUs, the Mediterranean *A. aerophoba* could be clearly distinguished. Based on co-occurrence, three prominent bacterial sponge symbionts belonging to the Chloroflexi and Nitrospinae were identified as potential sources of abundant halogenases. These results may thus contribute to explaining the origin of the numerous halogenated compounds discovered in *Aplysina* species.

## Supplementary Material

fiz108_Supplemental_File
