# Supplementary material for: Diversity of tryptophan halogenases in sponges of the genus Aplysina
Source: FEMS Microbiol Ecol. 2019 Jul 5;95(8):fiz108. doi: 10.1093/femsec/fiz108 (PMC6644159; doi:10.1093/femsec/fiz108)
Supplement: fiz108_Supplemental_File [file fiz108_supplemental_file.zip › Supplementary_Figure_Legends.docx]

# Supplementary Figure Legends

Supplementary Figure S1: Relative abundance of bacterial phyla in *Aplysina* species.

Supplementary Figure S2: Relative abundance of the overall 50 most abundant 16S rRNA gene OTUs in Aplysina species.

Supplementary Figure S3: Linear correlations between diversity metrics calculated for bacterial (B) 16S rRNA genes (x-axis) and halogenases (H) (y-axis).

Supplementary Figure S4: Heatmap showing significant (Spearman r>±0.5, p<0.05) correlation of relative halogenase abundances (columns) with the relative abundance of the overall 50 most prevalent bacterial OTUs (rows). Columns and rows are clustered by Euclidian distance.
