# Supplementary material for: Diversity of tryptophan halogenases in sponges of the genus Aplysina
Source: FEMS Microbiol Ecol. 2019 Jul 5;95(8):fiz108. doi: 10.1093/femsec/fiz108 (PMC6644159; doi:10.1093/femsec/fiz108)
Supplement: fiz108_Supplemental_File [file fiz108_supplemental_file.zip › TC_Supplementary_Table.docx]

# Supplementary Table:

Table S1: Summary of the closest matches of "non-halogenase" sequences in the UniProt database. The results of the top 10 most abundant sequences with a given protein name hit are displayed. (*) One out of several accession numbers are given as example.

| **Protein name** | **No. of sequences** | **%ID min.** | **%ID max.** | **Bitscore min.** | **Bitscore max.** | **UniProt accession** |
| --- | --- | --- | --- | --- | --- | --- |
| P-protein | 13 | 44.8 | 60.9 | 28.9 | 35.8 | O67085 |
| Prephenate dehydratase | 10 | 55.6 | 63 | 30.4 | 35.4 | P9WIC3 |
| Translation initiation factor IF-2 | 9 | 31.6 | 71.1 | 25.8 | 60.8 | Q1IIT3* |
| Divinyl chlorophyll a/b light-harvesting protein PcbE | 8 | 48 | 52 | 26.9 | 32.3 | Q46JW8 |
| Genome polyprotein | 8 | 30.3 | 43.5 | 27.7 | 39.7 | Q01901* |
| Repression factor of MSEs protein 1 | 7 | 40.9 | 40.9 | 25.8 | 27.7 | Q12192 |
| Valine--tRNA ligase | 7 | 27.1 | 44.8 | 25.4 | 35 | Q5E7U0* |
| 1,4-alpha-glucan branching enzyme GlgB | 6 | 42.2 | 70.6 | 25.8 | 32.3 | A6VP15* |
| Cartilage acidic protein 1 | 6 | 34.1 | 42.3 | 26.6 | 47.8 | Q0V9M0* |
| Cysteine desulfurase IscS | 6 | 37 | 46.5 | 27.7 | 107 | B8DZS1* |
|  | 1,237 | more rows | |  |  |  |
