# Supplementary figures and images for: Diversity of tryptophan halogenases in sponges of the genus Aplysina
Source: FEMS Microbiol Ecol. 2019 Jul 5;95(8):fiz108. doi: 10.1093/femsec/fiz108 (PMC6644159; doi:10.1093/femsec/fiz108)

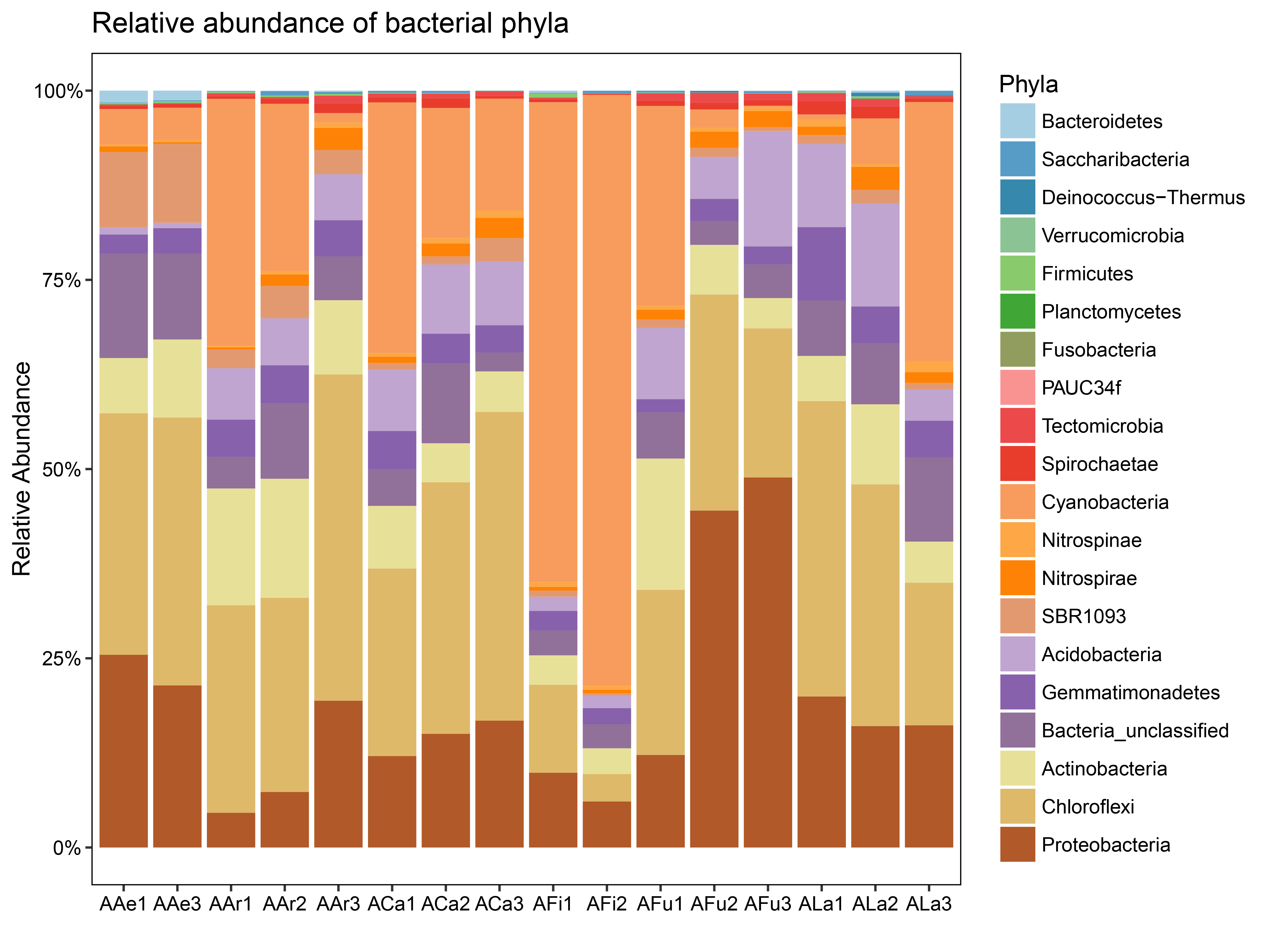

Supplement: fiz108_Supplemental_File [file fiz108_supplemental_file.zip › Sup_Figure_S1.tif]

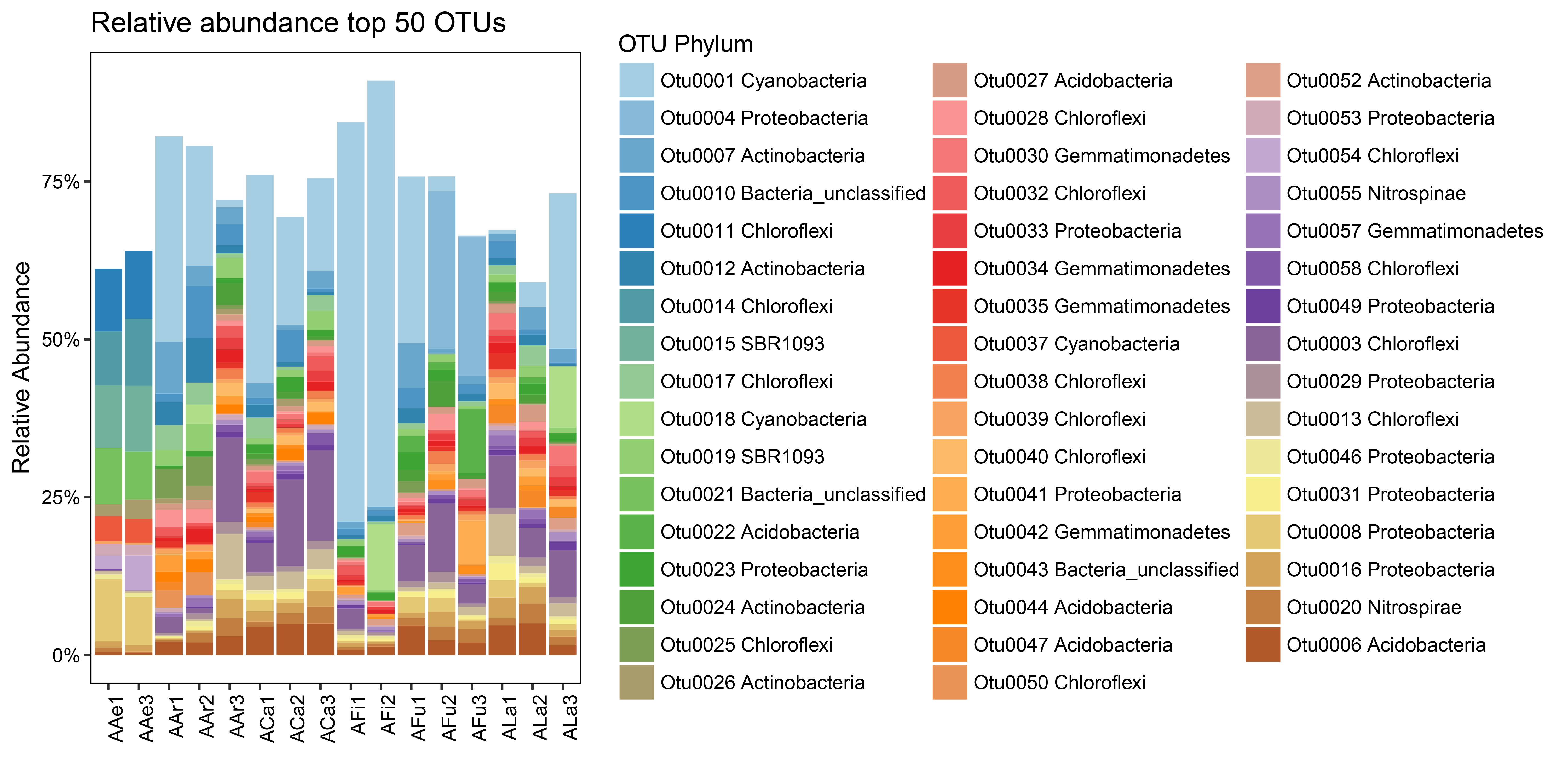

Supplement: fiz108_Supplemental_File [file fiz108_supplemental_file.zip › Sup_Figure_S2.tif]

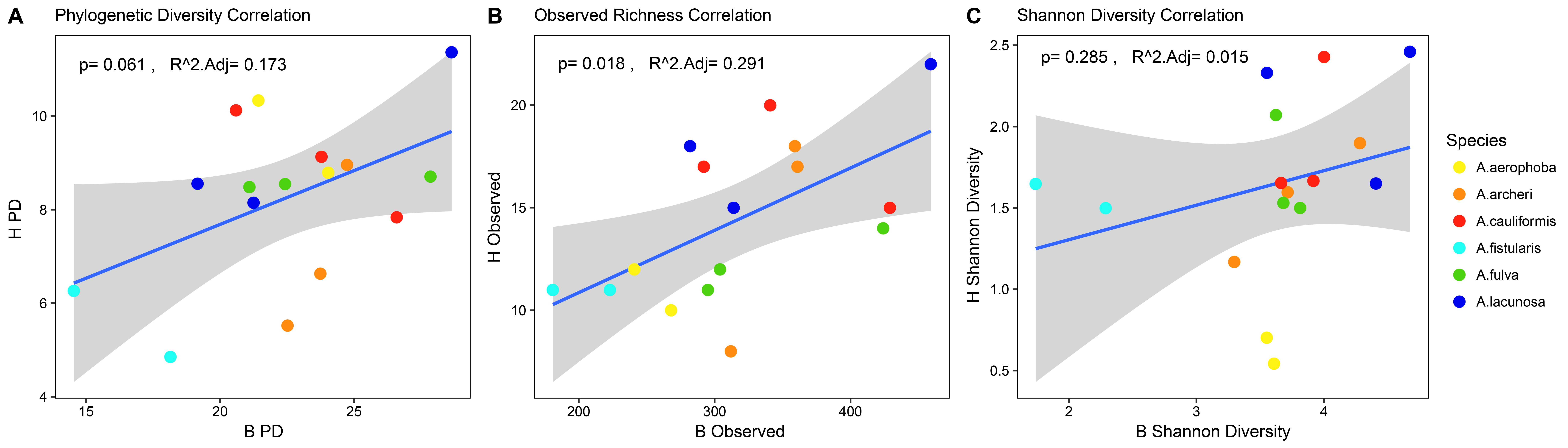

Supplement: fiz108_Supplemental_File [file fiz108_supplemental_file.zip › Sup_Figure_S3_corrected.tif]

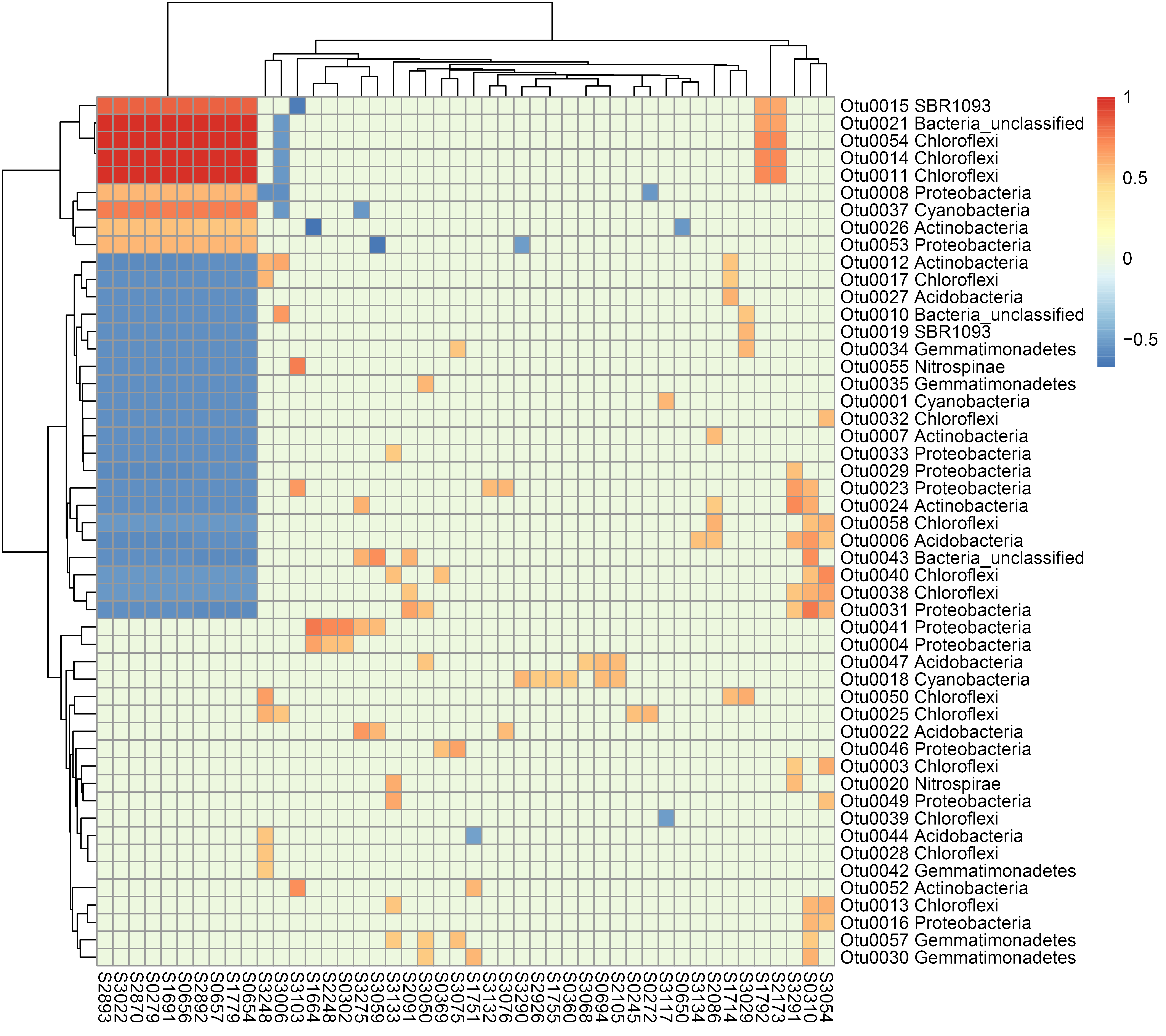

Supplement: fiz108_Supplemental_File [file fiz108_supplemental_file.zip › Sup_Figure_S4.tif]
